# Supplementary material for: Transcriptome Analysis Reveals Vimentin-Induced Disruption of Cell–Cell Associations Augments Breast Cancer Cell Migration
Source: Cells. 2022 Dec 13;11(24):4035. doi: 10.3390/cells11244035 (PMC9776984; doi:10.3390/cells11244035)
Supplement: Supplementary file 1 [file cells-11-04035-s001.zip › DEGs and enrichment analysis.zip!/DEGs and enrichment analysis/WT_VIMvsVector GO enrichment/GSEA/WT_VIM/SMAD_PROTEIN_SIGNAL_TRANSDUCTION/SMAD_PROTEIN_SIGNAL_TRANSDUCTION(GO_0060395).html]

Details for gene set SMAD\_PROTEIN\_SIGNAL\_TRANSDUCTION(GO:0060395)[GSEA]


|  || Dataset | fpkm.sample |
| Phenotype | sample.cls |
| Upregulated in class | WT\_VIM |
| GeneSet | SMAD\_PROTEIN\_SIGNAL\_TRANSDUCTION(GO:0060395) |
| Enrichment Score (ES) | 0.92745876 |
| Normalized Enrichment Score (NES) | 1.461754 |
| Nominal p-value | 0.0 |
| FDR q-value | 0.1629998 |
| FWER p-Value | 0.163 |
Table: GSEA Results Summary

  

Fig 1: Enrichment plot: SMAD\_PROTEIN\_SIGNAL\_TRANSDUCTION(GO:0060395)
  
Profile of the Running ES Score & Positions of GeneSet Members on the Rank Ordered List

  

| PROBE | DESCRIPTION   (from dataset) | GENE SYMBOL | GENE\_TITLE | RANK IN GENE LIST | RANK METRIC SCORE | RUNNING ES | CORE ENRICHMENT || 1 | ENSG00000101076 | HNF4A |  |  | 463 | Infinity | -0.0078 | Yes |
| 2 | ENSG00000026025 | VIM |  |  | 2388 | 1709.539 | 0.9275 | Yes |
| 3 | ENSG00000169071 | ROR2 |  |  | 3924 | 3.097 | 0.9032 | No |
| 4 | ENSG00000248329 | APELA |  |  | 4308 | 2.787 | 0.8983 | No |
| 5 | ENSG00000125378 | BMP4 |  |  | 6326 | 1.969 | 0.8653 | No |
| 6 | ENSG00000239306 | RBM14 |  |  | 6434 | 1.937 | 0.8646 | No |
| 7 | ENSG00000119699 | TGFB3 |  |  | 7011 | 1.815 | 0.8558 | No |
| 8 | ENSG00000139269 | INHBE |  |  | 7171 | 1.779 | 0.8542 | No |
| 9 | ENSG00000105329 | TGFB1 |  |  | 8097 | 1.609 | 0.8394 | No |
| 10 | ENSG00000102900 | NUP93 |  |  | 8372 | 1.566 | 0.8357 | No |
| 11 | ENSG00000101144 | BMP7 |  |  | 8436 | 1.555 | 0.8355 | No |
| 12 | ENSG00000112175 | BMP5 |  |  | 8527 | 1.544 | 0.8348 | No |
| 13 | ENSG00000175189 | INHBC |  |  | 8639 | 1.530 | 0.8338 | No |
| 14 | ENSG00000135414 | GDF11 |  |  | 8661 | 1.526 | 0.8343 | No |
| 15 | ENSG00000143799 | PARP1 |  |  | 8977 | 1.486 | 0.8298 | No |
| 16 | ENSG00000157110 | RBPMS |  |  | 9540 | 1.412 | 0.8211 | No |
| 17 | ENSG00000156466 | GDF6 |  |  | 9982 | 1.362 | 0.8144 | No |
| 18 | ENSG00000136999 | NOV |  |  | 10132 | 1.342 | 0.8127 | No |
| 19 | ENSG00000135100 | HNF1A |  |  | 10469 | 1.318 | 0.8077 | No |
| 20 | ENSG00000153162 | BMP6 |  |  | 10987 | 1.267 | 0.7997 | No |
| 21 | ENSG00000043143 | JADE2 |  |  | 11564 | 1.222 | 0.7906 | No |
| 22 | ENSG00000120693 | SMAD9 |  |  | 11578 | 1.220 | 0.7911 | No |
| 23 | ENSG00000183682 | BMP8A |  |  | 11823 | 1.198 | 0.7876 | No |
| 24 | ENSG00000156574 | NODAL |  |  | 11936 | 1.187 | 0.7864 | No |
| 25 | ENSG00000125931 | CITED1 |  |  | 12215 | 1.164 | 0.7824 | No |
| 26 | ENSG00000163083 | INHBB |  |  | 12379 | 1.150 | 0.7803 | No |
| 27 | ENSG00000157933 | SKI |  |  | 13763 | 1.045 | 0.7574 | No |
| 28 | ENSG00000107779 | BMPR1A |  |  | 14665 | 0.991 | 0.7427 | No |
| 29 | ENSG00000143869 | GDF7 |  |  | 14783 | 0.983 | 0.7413 | No |
| 30 | ENSG00000123999 | INHA |  |  | 14931 | 0.974 | 0.7394 | No |
| 31 | ENSG00000170345 | FOS |  |  | 15485 | 0.940 | 0.7305 | No |
| 32 | ENSG00000170365 | SMAD1 |  |  | 17010 | 0.857 | 0.7052 | No |
| 33 | ENSG00000108187 | PBLD |  |  | 17328 | 0.838 | 0.7003 | No |
| 34 | ENSG00000144959 | NCEH1 |  |  | 17787 | 0.811 | 0.6930 | No |
| 35 | ENSG00000113387 | SUB1 |  |  | 18679 | 0.761 | 0.6784 | No |
| 36 | ENSG00000175387 | SMAD2 |  |  | 19473 | 0.717 | 0.6654 | No |
| 37 | ENSG00000141646 | SMAD4 |  |  | 20399 | 0.665 | 0.6501 | No |
| 38 | ENSG00000164404 | GDF9 |  |  | 20490 | 0.661 | 0.6489 | No |
| 39 | ENSG00000116985 | BMP8B |  |  | 20676 | 0.651 | 0.6462 | No |
| 40 | ENSG00000176973 | FAM89B |  |  | 21678 | 0.597 | 0.6296 | No |
| 41 | ENSG00000064393 | HIPK2 |  |  | 21817 | 0.587 | 0.6276 | No |
| 42 | ENSG00000177606 | JUN |  |  | 22299 | 0.557 | 0.6197 | No |
| 43 | ENSG00000168874 | ATOH8 |  |  | 22425 | 0.549 | 0.6179 | No |
| 44 | ENSG00000018408 | WWTR1 |  |  | 22442 | 0.548 | 0.6180 | No |
| 45 | ENSG00000169359 | SLC33A1 |  |  | 22482 | 0.545 | 0.6176 | No |
| 46 | ENSG00000106799 | TGFBR1 |  |  | 22648 | 0.534 | 0.6151 | No |
| 47 | ENSG00000113441 | LNPEP |  |  | 22971 | 0.508 | 0.6100 | No |
| 48 | ENSG00000187391 | MAGI2 |  |  | 23278 | 0.489 | 0.6051 | No |
| 49 | ENSG00000113658 | SMAD5 |  |  | 23755 | 0.460 | 0.5973 | No |
| 50 | ENSG00000151136 | BTBD11 |  |  | 23845 | 0.451 | 0.5960 | No |
| 51 | ENSG00000130513 | GDF15 |  |  | 23873 | 0.449 | 0.5958 | No |
| 52 | ENSG00000166949 | SMAD3 |  |  | 24688 | 0.383 | 0.5822 | No |
| 53 | ENSG00000122641 | INHBA |  |  | 25001 | 0.353 | 0.5772 | No |
| 54 | ENSG00000153071 | DAB2 |  |  | 25884 | 0.260 | 0.5624 | No |
| 55 | ENSG00000125845 | BMP2 |  |  | 26066 | 0.243 | 0.5594 | No |
| 56 | ENSG00000092969 | TGFB2 |  |  | 26493 | 0.192 | 0.5523 | No |
| 57 | ENSG00000125965 | GDF5 |  |  | 30142 | 0.000 | 0.4906 | No |
| 58 | ENSG00000138379 | MSTN |  |  | 30390 | 0.000 | 0.4864 | No |
| 59 | ENSG00000263761 | GDF2 |  |  | 32181 | NaN | 0.4561 | No |
| 60 | ENSG00000152785 | BMP3 |  |  | 33601 | NaN | 0.4321 | No |
| 61 | ENSG00000266524 | GDF10 |  |  | 37324 | NaN | 0.3691 | No |
| 62 | ENSG00000130283 | GDF1 |  |  | 38106 | NaN | 0.3559 | No |
| 63 | ENSG00000164458 | TBXT |  |  | 44692 | NaN | 0.2444 | No |
| 64 | ENSG00000130385 | BMP15 |  |  | 45309 | NaN | 0.2340 | No |
| 65 | ENSG00000243709 | LEFTY1 |  |  | 48387 | NaN | 0.1819 | No |
| 66 | ENSG00000184344 | GDF3 |  |  | 51060 | NaN | 0.1366 | No |
| 67 | ENSG00000081051 | AFP |  |  | 52648 | NaN | 0.1098 | No |
| 68 | ENSG00000163217 | BMP10 |  |  | 53920 | NaN | 0.0883 | No |
| 69 | ENSG00000143768 | LEFTY2 |  |  | 58148 | NaN | 0.0167 | No |
Table: GSEA details
[plain text format]

  

Fig 2: SMAD\_PROTEIN\_SIGNAL\_TRANSDUCTION(GO:0060395)
  
Blue-Pink O' Gram in the Space of the Analyzed GeneSet

  

Fig 3: SMAD\_PROTEIN\_SIGNAL\_TRANSDUCTION(GO:0060395): Random ES distribution
  
Gene set null distribution of ES for
 **SMAD\_PROTEIN\_SIGNAL\_TRANSDUCTION(GO:0060395)**
